# Sleep apnoea headache in obstructive sleep apnoea syndrome patients presenting with morning headache: comparison of the ICHD-2 and ICHD-3 beta criteria

**DOI:** 10.1186/s10194-015-0540-6

**Published:** 2015-06-19

**Authors:** Keisuke Suzuki, Masayuki Miyamoto, Tomoyuki Miyamoto, Ayaka Numao, Shiho Suzuki, Hideki Sakuta, Akio Iwasaki, Yuji Watanabe, Hiroaki Fujita, Koichi Hirata

**Affiliations:** Department of Neurology, Dokkyo Medical University, 880 Kitakobayashi, Mibu, Shimotsuga, Tochigi, 321-0293 Japan; School of Nursing, Dokkyo Medical University, Tochigi, Japan; Department of Neurology, Dokkyo Medical University Koshigaya Hospital, Saitama, Japan

**Keywords:** Sleep apnoea headache, Morning headache, Obstructive sleep apnoea syndrome

## Abstract

**Background:**

Morning headache is associated with obstructive sleep apnoea syndrome (OSAS); however, OSAS patients present with various characteristics of morning headache, and they often do not fulfil the International Classification of Headache Disorders (ICHD)-2 criteria for “sleep apnoea headache”. The aims of this study were to assess the new ICHD-3 beta criteria for sleep apnoea headache in OSAS patients and to evaluate the differences with the ICHD-2.

**Methods:**

We conducted a cross-sectional survey regarding morning and sleep apnoea headaches that included 235 OSAS outpatients receiving continuous positive airway pressure (CPAP) treatment. The presence of morning headache was evaluated by reviewing the medical records before administration of CPAP treatment.

**Results:**

Of all of the OSAS patients, 48 (20.4 %) reported morning headaches. Of the 48 patients with morning headaches, 29 (60.4 %) and 39 (81.3 %) fulfilled the ICHD-2 and ICHD-3 beta criteria for sleep apnoea headache, respectively. The increased frequency of individuals who qualified for diagnosis was likely attributable to the extension of headache duration from 30 min to 4 h. The severity of OSAS was not associated with the presence of sleep apnoea headache.

**Conclusions:**

The utilisation of ICHD-3 beta criteria is clinically useful for diagnosing sleep apnoea headache in patients with OSAS. Applying the ICHD-3 beta criteria was of clinical significance when considering the marked response of these headaches to CPAP therapy. The cause of sleep apnoea headache remains to be elucidated.

## Background

Morning headache has been recognised as an obstructive sleep apnoea syndrome (OSAS)-related symptom, in addition to daytime sleepiness and snoring. However, previous reports have attributed morning headaches to different causes: nocturnal desaturation, hypercapnia or OSAS severity [1]; non-OSAS sleep disorders or non-specific symptoms related to primary/secondary headaches [2]; and psychiatric disorders, such as depression [3]. The appropriate management of OSAS in patients with chronic headache is also clinically important because in some patients, continuous positive airway pressure (CPAP) treatment is effective at improving their headaches [4]. In the International Classification of Headache Disorders, second edition, (ICHD-2) [5], headaches upon awakening in the morning and during sleep related to OSAS were included as “sleep apnoea headache” (Table 1). The characteristics of sleep apnoea headaches are bilateral pain that is pressing rather than pulsating, short duration (<30 min) and a frequency greater than 15 days per month. They resolve within 72 h after effective treatment of OSAS.

**Table 1:** Diagnostic criteria for sleep apnoea headache according to the ICHD-2 (code 10.1.3) and the percentage of OSAS patients

Diagnostic criteria	Morning headache (*n* = 48)
A. Recurrent headache with at least one of the following characteristics and fulfilling criteria C and D (sleep apnoea headache):	*n* = 29 (60.4 %)
1. occurs on > 15 days per month	*n* =12 (25.0 %)
2. bilateral, pressing quality and not accompanied by nausea, photophobia or phonophobia	*n* = 20 (41.7 %)
3. each headache resolves within 30 min	*n* = 15 (31.3 %)
B. Sleep apnoea (Respiratory Disturbance Index ≥ 5) demonstrated by overnight polysomnography	*n* = 48 (100 %)
C. Headache is present upon awakening	*n* = 48 (100 %)
D. Headache ceases within 72 h and does not recur after effective treatment of sleep apnoea	*n* = 39 (81.3 %)

We often encounter OSAS patients who present with various characteristics of morning headache that often do not fulfil the ICHD-2 criteria for “sleep apnoea headache”; however, most of their morning headaches respond well to CPAP treatment. Likewise, some investigators have suggested that not all characteristics and frequencies of morning headache fit the definition of “sleep apnoea headache” [1, 6]. In light of these observations, the definition of sleep apnoea headache has been slightly modified in the latest ICHD-3 beta version (3 beta) [7], which defines sleep apnoea headache as a morning headache, usually bilateral and with a duration of less than 4 h, caused by sleep apnoea with no time restriction on headache improvement following appropriate therapy for OSAS (Table 2). However, no studies have been designed to compare these two sets of criteria for diagnosing sleep apnoea headache.

**Table 2:** Diagnostic criteria for sleep apnoea headache according to the ICHD-3 beta (code 10.1.4) and the percentage of OSAS patients

Diagnostic criteria	Morning headache (*n* = 48)
A. Headache present on awakening after sleep and fulfilling criterion C (Sleep apnoea headache)	*n* = 39 (81.3 %)
B. Sleep apnoea (apnoea-hypopnoea index 5) has been diagnosed	*n* = 48 (100 %)
C. Evidence of causation demonstrated by at least two of the following:	*n* = 39 (81.3 %)
1. Headache has developed in temporal relation to the onset of sleep apnoea	
2. Either or both of the following:	
a) Headache has worsened in parallel with worsening of sleep apnoea	
b) Headache has significantly improved or remitted in parallel with improvement in or resolution of sleep apnoea	
3. Headache has at least one of the following three characteristics:	
a) Recurs on > 15 days per month	*n* = 12 (25.0 %)
b) All of the following:	*n* = 20 (41.7 %)
(i) bilateral location	
(ii) pressing quality	
(iii) not accompanied by nausea, photophobia or phonophobia	
c) Resolves within 4 h	*n* = 25 (52.1 %)
D. Not better accounted for by another ICHD-3 diagnosis	

The aims of this study were to assess the new ICHD-3 beta criteria for sleep apnoea headache in OSAS patients and to evaluate the differences with the ICHD-2 criteria.

## Methods

**Fig. 1:**
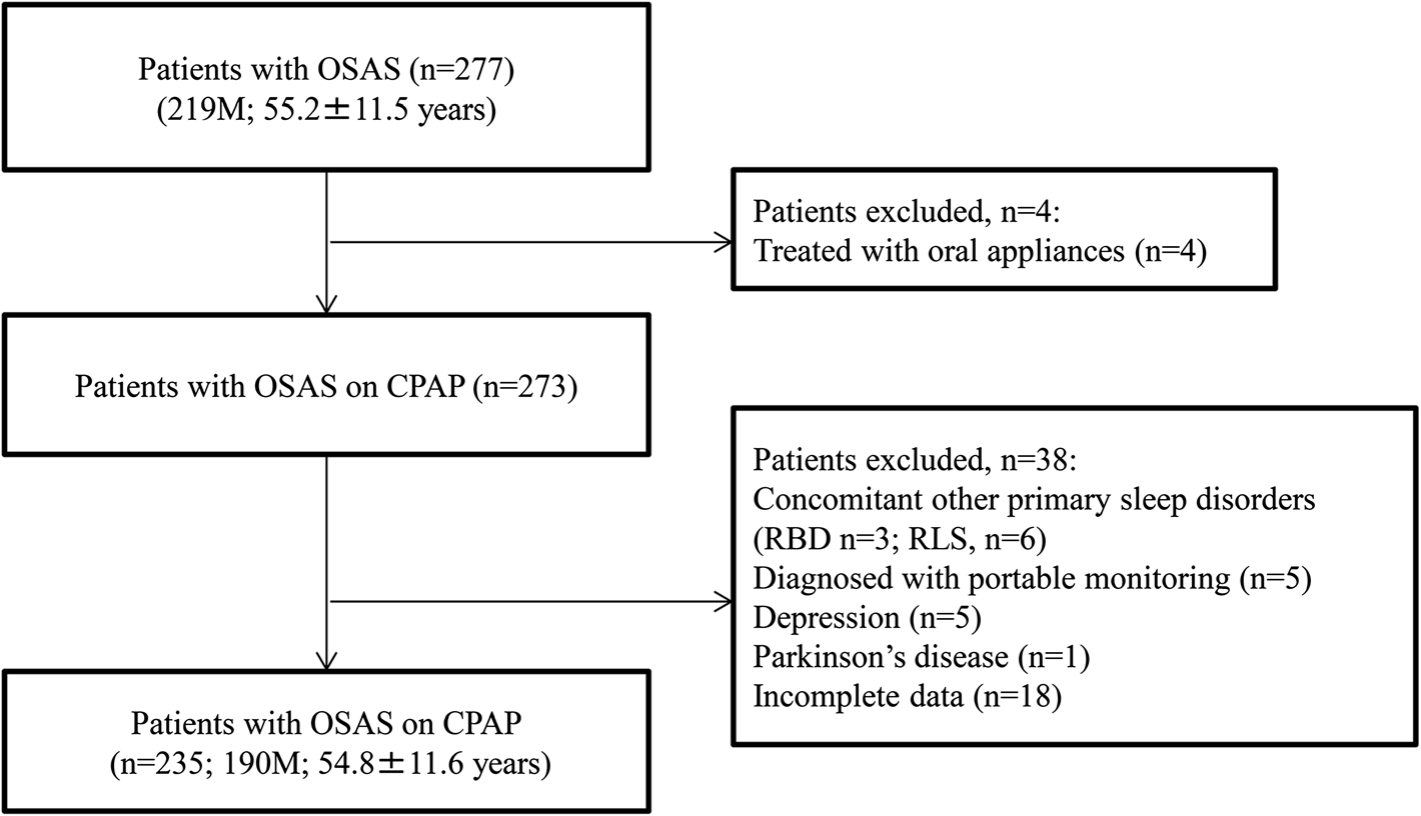
Flow chart of the patient selection process. RBD, rapid eye movement sleep behaviour disorder; RLS, restless legs syndrome

We conducted a cross-sectional survey of morning and sleep apnoea headaches between July 2012 and March 2013. This study was approved by the Dokkyo Medical University institutional review board and conducted in accordance with the Declaration of Helsinki. All participants provided written informed consent to participate in the study. Patients with OSAS who were regularly seen in our outpatient clinic at the Department of Neurology, Dokkyo Medical University and whose conditions were at stable for at least 3 months were included in this study. Figure 1 displays the flowchart for this study. Initially, 277 consecutive outpatients with OSAS (219 men, 58 women; mean age, 55.2 ± 11.5 years) were enrolled. Patients receiving oral appliance treatment (*n* = 4); patients with other comorbid primary sleep disorders, such as rapid eye movement sleep behaviour disorders (*n* = 3) and restless legs syndrome (RLS) (*n* = 6); and patients with neurodegenerative diseases and psychiatric disorders were excluded from the study. Patients with OSAS diagnosed by portable monitoring were excluded (*n* = 5). Finally, 235 stable outpatients with OSAS who were receiving CPAP therapy (190 men, 45 women; mean age, 54.8 ± 11.6 years) were included in this study (Fig. 1).

OSAS was diagnosed on the basis of a clinical interview and examination and in-laboratory full-night polysomnography (PSG), according to the International Classification of Sleep Disorders, second edition (ICSD-2) [8]. Obstructive sleep apnoea was defined as the cessation of airflow for at least 10 s in the presence of respiratory effort. Hypopnea was defined as a greater than 50 % reduction of airflow for at least 10 s, a reduction in breathing of less than 50 %, greater than 3 % oxygen desaturation (ODI) or arousal for more than 10 s. ODI was calculated according to the number of oxygen desaturation values greater than 3 % per hour of sleep. The apnoea-hypopnoea index (AHI) was calculated as the average of the total number of apnoea and hypopnoea episodes per hour of sleep. No patients had central apnoea index greater than 5. Data were extracted from PSG records and medical records. Of all of the patients, 225 patients (95.7 %) had an AHI of 20 or greater. Data on daily CPAP usage and residual AHI after CPAP treatment were obtained from CPAP memory cards for at least 2 consecutive months after CPAP introduction. Acceptable CPAP compliance (% CPAP usage > 4 h/day) was defined when the patient used CPAP > 4 h per night for > 70 % of nights over at least 2 consecutive months. CPAP responders were defined by residual AHI < 10.

All of the patients were asked about the presence and characteristics (localisation, quality and intensity) of morning headache (headache upon waking in the morning) by MM or KS during a face-to-face interview. In addition, through review of the medical records before administration of CPAP treatment, the presence of morning headaches was confirmed. Patients who had a headache after a nap or during sleep but not upon waking were not considered to have “morning headache” in this study. If these patients reported having morning headache, their headaches were classified as migraines, tension-type headaches, cluster headaches or others (unclassified type) according to the ICHD-2 criteria. For headache characteristics, only headaches occurring upon waking were assessed. Then, morning headaches were assessed to determine whether they fulfilled the criteria for “sleep apnoea headache” according to ICHD-3 beta (sleep apnoea headache [ICHD-3 beta]) criteria [7], and the differences with the ICHD-2 criteria [5] were evaluated. Regarding the effectiveness of CPAP treatment on morning headache, improvement of morning headache after CPAP therapy was defined when headaches ceased and did not recur after CPAP treatment.

Sleep disturbance, sleep quality and sleep habits were assessed using the Japanese version of the Pittsburgh Sleep Quality Index (PSQI). The following seven component scores of the PSQI were also evaluated [9]: C1, sleep quality; C2, sleep latency; C3, sleep duration; C4, habitual sleep efficiency; C5, sleep disturbances; C6, use of sleeping medications; and C7, daytime dysfunctions. Daytime sleepiness was evaluated with the use of the Japanese version of the Epworth Sleepiness Scale (ESS) [10, 11]. The Beck Depression Inventory-II (BDI-II) was employed to assess depressive symptoms [12].

### Statistical analysis

The Mann–Whitney U-test or unpaired t-test was used where appropriate to compare continuous variables, and the chi-squared or Fisher’s exact test was performed to compare the differences in frequencies between two groups. The significance of the differences was defined using a two-tailed t-test with *p* < 0.05. A commercially available software package (IBM SPSS Statics 21.0, Tokyo, Japan) was used for the statistical analyses.

## Results

Of 235 OSAS patients, 48 (20.4 %) reported morning headaches. Of the 48 patients with morning headache, 25.0 % had a headache frequency greater than 15 days per month, 41.7 % had pressing and bilateral headache not accompanied by nausea, photophobia or phonophobia, 31.3 % had a headache of short duration (<30 min), and 81.3 % reported improvement in their headaches after CPAP treatment. Overall, 29 patients (60.4 %) fulfilled the ICHD-2 diagnostic criteria for sleep apnoea headache (Table 1). When the ICHD-3 beta criteria for sleep apnoea headache were used, 81.3 % patients with morning headache fulfilled the sleep apnoea headache criteria (Table 2). In particular, 10 patients with morning headache who responded well to CPAP treatment did not fulfil the ICHD-2 criteria because their headache durations were longer than 30 min. However, when the ICHD-3 beta criteria were applied, all of these patients were diagnosed with “sleep apnoea headache”.

Table 3 displays the clinical characteristics of the patients with sleep apnoea headache, according to the ICHD-2 and ICHD-3 beta criteria. Patients with sleep apnoea headache were younger and had higher BMI and more severe depressive symptoms than those without sleep apnoea headache. However, CPAP adherence and PSG parameters, such as AHI, 3 % ODI and arousal index, were similar between the groups.

**Table 3:** Comparison of clinical features of patients with and without sleep apnoea headache

	ICHD-2		*p* value	ICHD-3 beta		*p* value
	Sleep apnoea headache (*n* = 29)	No sleep apnoea headache (*n* = 206)		Sleep apnoea headache (*n* = 39)	No sleep apnoea headache (*n* = 196)	
Age (years)	50.4 ± 10.6	55.4 ± 11.6	**0.028**	49.3 ± 10.1	55.9 ± 11.6	**0.0012**
BMI (kg/m^2^)	31.7 ± 10.0	28.2 ± 7.3	0.084	31.5 ± 9.0	28.1 ± 7.4	**0.011**
Sex (M/F)	21/8	169/37	0.22	30/9	160/36	0.50
Education (years)	14.6 ± 2.9	13.3 ± 2.7	**0.028**	14.8 ± 3.4	13.2 ± 2.6	**0.0073**
Caffeine (cup/day)	2.6 ± 1.9	2.9 ± 2.6	0.54	2.7 ± 1.8	2.9 ± 2.6	0.73
Smoking, n (%)			0.28			0.17
Never	9 (31.0)	87 (42.2)		13 (33.3)	83 (42.3)	
Past	12 (41.4)	85 (41.3)		15 (38.5)	82 (41.8)	
Current	8 (27.6)	34 (16.5)		11 (28.2)	31 (15.8)	
Alcohol, n (%)			**0.040**			**0.0057**
Never	8 (27.6)	70 (34.0)		11 (28.2)	67 (34.2)	
< 1 day/week	14 (48.3)	50 (24.3)		19 (48.7)	45 (23.0)	
1-2 days/week	4 (13.8)	22 (10.7)		5 (12.8)	21 (10.7)	
3-5 days/week	2 (6.9)	27 (13.1)		3 (7.7)	26 (13.3)	
6-7 days/week	1 (3.4)	37 (18.0)		1 (2.6)	37 (18.9)	
AHI (/h) (before CPAP treatment)	51.6 ± 22.6	55.3 ± 25.2	0.46	56.6 ± 24.9	54.5 ± 24.9	0.63
3 % ODI (/h)	41.5 ± 23.7	45.4 ± 26.6	0.47	46.9 ± 26.7	44.5 ± 26.2	0.61
Arousal index (/h)	47.0 ± 17.5	56.1 ± 48.1	0.41	51.3 ± 20.6	55.7 ± 48.9	0.66
Morning headache	29 (100)	19 (9.2)	**<0.001**	39 (100)	9 (4.6)	**<0.001**
ESS (before CPAP treatment)	8.7 ± 4.7	8.5 ± 5.2	0.88	8.6 ± 4.6	8.5 ± 5.3	0.90
PSQI global score	5.8 ± 2.6	5.1 ± 3.1	0.23	5.9 ± 2.9	5.0 ± 3.0	0.085
PSQI component score						
C1, Sleep quality	1.4 ± 0.6	1.1 ± 0.7	0.052	1.3 ± 0.7	1.1 ± 0.7	0.064
C2, Sleep latency	0.8 ± 1.0	0.6 ± 0.9	0.52	0.8 ± 1.0	0.6 ± 0.9	0.28
C3, Sleep duration	1.4 ± 0.9	1.1 ± 0.9	0.055	1.4 ± 0.9	1.1 ± 0.9	0.062
C4, Habitual sleep efficiency	0.3 ± 0.7	0.2 ± 0.7	0.48	0.3 ± 0.6	0.2 ± 0.7	0.80
C5, Sleep disturbances	1.0 ± 0.6	0.9 ± 0.5	0.20	0.9 ± 0.6	0.9 ± 0.5	0.39
C6, Use of sleeping medication	0.3 ± 0.8	0.4 ± 1.0	0.54	0.5 ± 1.0	0.4 ± 1.0	0.51
C7, Daytime dysfunction	0.7 ± 0.8	0.7 ± 0.9	0.62	0.7 ± 0.8	0.7 ± 0.9	0.93
BDI-II	11.4 ± 6.9	9.1 ± 7.3	0.10	11.5 ± 7.1	8.9 ± 7.3	**0.042**
Residual AHI after CPAP treatment (/h)^a^	3.5 ± 2.3	3.7 ± 2.5	0.67	3.6 ± 2.5	3.7 ± 2.4	0.88
CPAP responders (residual AHI < 10), n (%)	29 (100)	202 (98.1)	1.0	38 (97.4)	193 (98.5)	0.65
CPAP treatment period (/m)	29.0 ± 29.4	36.4 ± 29.0	0.20	29.2 ± 28	36.8 ± 29.2	0.14
Daily CPAP use (h)	5:03 ± 1:24	5:04 ± 1:38	0.98	4:45 ± 1:33	5:07 ± 1:36	0.19
CPAP usage > 4 h/d, n (%)	16 (55.2)	106 (51.5)	0.71	19 (48.7)	103 (52.6)	0.66

The data are shown as n (number or %) or mean ± SD. Statistically significant values (*p* < 0.05) are shown in bold

*AHI* apnoea-hypopnoea index, *BDI-II* Beck Depression Inventory-II, *CPAP* continuous positive airway pressure, *ESS* Epworth Sleepiness Scale, *ODI* oxygen desaturation index, *PSQI* Pittsburgh Sleep Quality Index

^a^Residual AHI was obtained from CPAP memory cards

OSAS patients with morning headache (*n* = 48) had the following characteristics: migraine, 12 (25.0 %); tension-type headache, 19 (39.6 %); cluster headache, 1 (2.1 %); and others (unclassified), 16 (33.3 %). Of the 12 patients with migraine characteristics, 7 (58.3 %) and 8 (66.7 %) had a diagnosis of sleep apnoea headache according to the ICHD-2 and ICHD-3 beta criteria, respectively. In contrast, of the 19 patients with tension-type headache characteristics, 12 (63.2 %) and 16 (84.2 %) had a diagnosis of sleep apnoea headache according to the ICHD-2 and ICHD-3 beta criteria, respectively. Of OSAS patients with unclassified types of morning headache (*n* = 16), 9 (56.3 %) and 14 (87.5 %) had a sole diagnosis of sleep apnoea headache according to the ICHD-2 and ICHD-3 beta criteria, respectively.

## Discussion

To the best of our knowledge, this study is the first to compare the sensitivity of the ICHD-3 beta criteria for “sleep apnoea headache” in OSAS patients with morning headaches. In our study, we assessed headaches upon awakening in the morning but not headaches upon awakening during sleep or after a nap because the ICHD-3 beta criteria clearly state that a sleep apnoea headache is a morning headache caused by sleep apnoea.

This study demonstrated that 60.4 % and 81.3 % of patients with morning headache met the criteria for “sleep apnoea headache” established by ICHD-2 and ICHD-3 beta, respectively (Tables 1 and 2). The increased frequency of individuals who qualified for diagnosis was likely attributable to the extension of headache duration from 30 min to 4 h. This modification had clinical significance, as demonstrated in this study. Out of all morning headaches (*n* = 48), 81.3 % (*n* = 39) responded to CPAP therapy, and 19.7 % (*n* = 10) of the CPAP-treatable morning headaches did not fulfil the ICHD-2 criteria, although they all fulfilled the ICHD-3 beta criteria for “sleep apnoea headache”. Previous studies have demonstrated that 80-90 % of morning headaches improved after appropriate treatment (CPAP or uvulopalatopharyngoplasty) for OSAS [1, 13]. Among habitual snorers, 32 % of all morning headaches lasted less than 30 min [6]; in our study of OSAS patients, 31.3 % of all morning headaches lasted less than 30 min. In other studies, the proportions of morning headaches lasting less than 1 h were 26.3 % [14] and 26.7 % [1] in OSAS patients. In our study, the proportion of morning headaches resolving within 4 h, that is, the duration specified by the ICHD-3 beta criteria, increased to 52.1 %.

Kristiansen et al. [15] recently reported an increased frequency of morning headache in participants with OSAS (11.8 %) compared with those without OSAS (4.16 %) in the general population. The frequency of morning headache (20.4 %) in our study was lower than previously reported in patients with OSAS (18-60 %) [1–3, 14, 16–18]. The proportion of total OSAS patients in this study who had sleep apnoea headache was 12.3 % and 16.6 % based on ICHD-2 and ICHD-3 beta, respectively; similarly, the latest review reports a prevalence of sleep apnoea headache of 12-18 % in the middle-aged population [19]. Göder et al. [20] compared PSG recordings from the nights before morning headaches with those from nights that were not followed by morning headaches in patients with OSAS, and they showed that the occurrence of a morning headache was associated with decreases in total sleep time, sleep efficiency and the amount of rapid eye movement sleep as well as with an increase in awake time during the preceding night. Oxygen desaturation and AHI did not differ when nights with and without subsequent morning headaches were compared. In our previous study including 36 OSAS patients, PaCO_2_ values upon waking did not significantly differ between patients with and without morning headaches [21]. In contrast, Aldrich and Chauncey [2] evaluated the frequency and characteristics of morning headaches in various sleep disorders, including OSAS, but they failed to find a significant association between morning headaches and OSAS. The possible mechanisms of sleep apnoea headache include repeated respiratory events, nocturnal hypoxia, hypercapnia-induced vasodilation and increased intracranial pressure [3, 13, 22]. Our study did not support a role of nocturnal hypoxia in the development of sleep apnoea headaches or morning headaches in OSAS patients because the PSG parameters, including AHI and nocturnal oxygen desaturation, did not differ depending on whether the patient had sleep apnoea headaches or morning headaches.

Another important finding in our study is that OSAS patients with morning headache frequently exhibited characteristics of migraine and tension-type headache. Furthermore, a significant number of morning headache patients having characteristics of migraine and tension-type headaches were diagnosed as having sleep apnoea headache. This finding suggests that for patients with OSAS, CPAP therapy can effectively treat not only morning headaches with tension-type features but also morning headaches with migraine-like features. By contrast, Chen et al. [6] found that morning headaches were more strongly associated with migraines (adjusted odds ratio, 6.3) than with OSAS (adjusted odds ratio, 2.6) in habitual snorers. The authors suggested that for habitual snorers, managing migraines and insomnia may be more helpful for morning headaches than managing OSAS. In the study including 4759 patients with OSAS from the Taiwan Longitudinal Health Insurance Database, OSAS patients had a higher likelihood of developing tension-type headache than did patients without OSAS [23]. However, the authors did not assess the presence of morning headache and whether tension-type headache was associated with morning headache. Johnson et al. [4] reported that of 82 chronic headache patients with migraine or tension-type headache or both, 63 % had concomitant OSAS.

The limitations of our study included the cross-sectional study design and the lack of a healthy control group. In this study, all of the OSAS patients were under CPAP treatment; therefore, the headache frequencies reported by the patients might have been underestimated due to the efficacy of CPAP therapy. However, the strength of this study is that sleep apnoea headaches were diagnosed after a confirmation of the effectiveness of CPAP on headaches, which could eliminate other headaches that mimicked sleep apnoea headaches; in the previous studies, morning headaches were evaluated before confirming the effectiveness of CPAP on headaches [6], or the CPAP effects on sleep apnoea headaches were evaluated in a limited number of OSAS patients [1, 3, 13]. In our study, most of the patients had an AHI of 20 or greater, likely because we selected stable OSAS patients receiving CPAP therapy who visited our outpatient clinic every month, and in Japan, CPAP use is generally restricted to OSAS patients with AHI ≥ 20. Further investigations are needed to evaluate sleep apnoea headache in OSAS patients with AHI < 20. A further prospective study is required to assess the associations between morning headaches and OSAS.

## Conclusions

The utilisation of ICHD-3 beta criteria is clinically useful for diagnosing sleep apnoea headache in patients with OSAS. The increased frequency of individuals who qualified for diagnosis of sleep apnoea headache is certainly of clinical significance when considering the marked response to CPAP therapy. Our study also showed that sleep apnoea headache was not related to OSAS severity. The cause of sleep apnoea headache remains to be elucidated.

## Abbreviations

AHI: Apnoea-hypopnea index
BDI-II: Beck Depression Inventory-II
CPAP: Continuous positive airway pressure
ESS: Epworth Sleepiness Scale
ICHD: International Classification of Headache Disorders
ODI: Oxygen desaturation
OSAS: Obstructive sleep apnoea syndrome
PSQI: Pittsburgh Sleep Quality Index
RBD: Rapid eye movement sleep behaviour disorder
RLS: Restless legs syndrome
